# Relative efficiency and productivity: a preliminary exploration of public hospitals in Beijing, China

**DOI:** 10.1186/1472-6963-14-158

**Published:** 2014-04-06

**Authors:** Hao Li, Siping Dong, Tingfang Liu

**Affiliations:** 1 School of Public Health\Global Health Institute, Wuhan University, Wuhan 430071, China; 2 Laboratorio Management e Sanità, Istituto di Management, Scuola Superiore Sant’Anna, Pisa 56127, Italy; 3 National Institute of Hospital Administration, Ministry of Health of P.R.C, Beijing 100191, China; 4 School of Political Science and Public Administration, Wuhan University, Wuhan 430072, China; 5 Institute for Hospital Management, Tsinghua University, Beijing 100084, China

**Keywords:** Public hospital, Total factor productivity, Technological change, Technical efficiency, Scale efficiency, Performance evaluation, China

## Abstract

**Background:**

Third-grade hospitals in Beijing have been rapidly developing in capacity and scale for many years. These hospitals receive a large number of patients, and ensuring their efficient operation is crucial in meeting people’s healthcare needs. In this context, a study of their relative efficiency and productivity would be helpful to identify the driving factors and further improve their performance.

**Methods:**

After a review of literature, the current numbers of open beds and employees were selected as input variables. The number of outpatient and emergency visits and the number of discharged patients were selected as output variables. A total of 12 third-grade Class A general public hospitals in Beijing were selected for a preliminary study. The panel data from 2006–2009 were collected by the National Institute of Hospital Administration, Ministry of Health of P.R. China. Descriptive analysis and data envelopment analysis were used to analyze the data using Stata 10.0 and DEAP(V2.1) software.

**Results:**

In the 2006–2009 period, descriptive results show that sample hospitals continuously expanded their capacity and scale, with growth rate of total revenue being the highest among all variables. The DEA results show that the average annual growth rate of productivity was 26.7%, and the rates were 47.3%, 21.3% and 13.8% respectively for two consecutive years. The average annual growth rate of technological change was 28.3%, and the rates were 49.4%, 21.5% and 16.4% respectively for two consecutive years. The average annual growth rate of technical efficiency change was -1.3%, and the rates were -1.4%, -0.02% and -2.2% respectively for two consecutive years.

**Conclusions:**

The sample hospitals in Beijing experienced substantial productivity growth, but annual growth rates were declining. Substantial technological change was the main contributor to the growth. Although some hospitals exhibited improvements in technical efficiency, there was a slight decline in general. To improve overall efficiency and productivity, both government and hospitals need to further drive positive technological change, technical change, and allocative efficiency of public hospitals. More empirical studies are needed to include more hospitals of all three grades at a larger scale.

## Background

China has emphasized industrial development and highlighted economic achievements since 1978, while the development in health care has long been lagged behind [1]. Although both income and healthcare needs increased, patients are faced with high healthcare cost and low accessibility to good quality care [2]. Since 1989, public hospitals in China have been accredited into three different grades classification system based on hospital functions, missions, facilities, professional construction, healthcare quality and safety, scientific management, etc [3]. The highest grade, third-grade hospitals are further classified into four classes (Top, A, B and C) according to their accreditation scores. All third-grade hospitals shall have more than 500 beds. The aforementioned accreditation system rendered many hospitals compete for capacity and scale to meet the higher grades’ standards [4]. Due to issues found in these competing practices, such accreditation system was suspended in 1998 by the Ministry of Health of China. However, the public generally would still judge a hospital by their accreditation grades and prefer third-grade Class hospitals for primary and hospital care. Consequently, third-grade hospitals receive a higher number of outpatients and inpatients. In this context, third-grade hospitals have incentives to continuously expand their capacity and scale to accommodate more patients, resulting in higher revenue and profit [5].

However, the high patient volume and scale do not necessarily correspond to efficient operation for these third-grade public hospitals. Indeed, many studies have found that efficiency and productivity of public hospitals still need improvements [6-12]. Pang and Wang [6] studied efficiency and productivity of 22 third-grade hospitals in 8 provincial cities and 1 municipality of China in the 2006–2007 period. They found that 63.6% productivity change can be explained by technological change and 16.6% productivity change can be explained by technical efficiency change. In Southeast China, Ng [12] made an efficiency study of 463 hospitals in Guangdong province between 2004 and 2008. She found that efficiency and productivity growth were deteriorating as technology progress. How about the current situation of hospitals in Beijing? It is well known that Beijing, as China’s capital city, not only receive the most benefits available from certain government policies, but also has the most competitive means in attracting capitals and human resources. This means that, the results of efficiency and productivity growth among public hospitals in Beijing may be different from other regions.

The purpose of this study is to obtain preliminary evidences for potential driving factors of relative efficiency and productivity in third-grade public hospitals in Beijing, and to identify effective ways for both government and hospitals to improve overall efficiency and productivity.

## Methods

### A review of approach

Concerning efficiency and productivity measurement, non parametric data envelopment analysis (DEA) and parametric stochastic frontier analysis (SFA) have been widely applied [13,14]. In SFA, a function of the efficient frontier needs to be constructed, while in DEA production frontier can be estimated according to observations of a group of inputs and outputs without constructing a function [15]. In many cases, allocated proportion and price information of production elements in health care services provided are difficult to acquire, while it is easier to obtain data of inputs and outputs in numbers. In this case the DEA is a popular tool.

There are many DEA models in the literature, among which three classical ones have been widely applied to measure relative efficiency and productivity: CCR, BCC and Malmquist Index. The CCR model was proposed by Charnes, Cooper and Rhodes [16], which is input oriented based on hypothesis of constant returns to scale (CRS). This model is suitable for efficiency and productivity study of multi-input and multi-output production units. The CCR model applies when all decision-making units (DMUs) are operating at optimum scale, which may be subject to incomplete competition, external constraints, financial conditions, etc. However, when DMUs are not operating at optimum scale, results of measured technical efficiency from CCR model may be altered by scale efficiency. Later Banker, Charnes and Cooper [17] proposed the BCC model, in which the CRS hypothesis was extended to variable returns to scale (VRS). In contrast, the VRS model can incorporate the impact of scale efficiency in measurement of technical efficiency.

Both aforementioned two models can be applied for single time period analysis (static state) of efficiency and productivity measurement. In contrast, the Malmquist index can be applied not only for multi-inputs and multi-outputs without price information, but also for single period and multi-period analysis [18]. This concept was proposed by Malmquist [19], and Caves et al. [20] used it to measure changes in a production unit’s efficiency in transforming inputs into outputs from time t to time t + 1. The index can be expressed using various distance functions. Total Factor Productivity (TFP) can be decomposed into changes in efficiency and changes in technology, allowing to measure their respective changes [20-22]. Assuming CRS in the DEA, the efficiency change can be further decomposed into pure efficiency change and scale efficiency change [22]. Any estimated efficiency differences assuming VRS and CRS respectively can reflect scale efficiency, which is computed as ratio between CRS VRS technical efficiency scores. In Malmquist Index, five indices can be generated: Technical Efficiency Change (TEC), Technological Change (TC), Pure Technical Efficiency Change (PTEC), Scale Efficiency Change (SEC) and Total Factor Productivity Change (TFPC). Many researches on how these indices are scored using linear programming are available [23,24]. If the score is greater than 1, efficiency/productivity has improved; if the score equals to 1, efficiency/productivity remains unchanged; if the score is less than 1, efficiency/productivity has declined.

### Instruments

In order to effectively measure relative efficiency and productivity of public hospitals in Beijing, it is crucial to first select a group of appropriate input and output variables. Table 1 lists these variables from journal papers published in Chinese [6-10]. It can be seen that most of Chinese researchers have applied a mix of variables, including monetary variables (fixed asset, expenditure, revenue), volume variables (actual number of open beds, number of employees), and ratio variables (bed occupation rate, number of stays in hospitals, etc.). The combined adoption of these variables may introduce double counting into efficiency and productivity analysis.

**Table 1:** DEA input and output variables

**Category**	**Variables**	**Definition**
Inputs	Actual number of open beds	The number of available bed days divided by the number of days in a year
	Number of employees	Registered employees at the end of year, excluding retirees and temporary staff
	Fixed assets	Tangible assets having been used more than one year with values amounting to specific standards and with their original physical attributes not changed
	Total expenditure	Capital consumption and loss in the process of service provision and other activities, including healthcare expenditure, drug and medicine expenditure, special financial expenditure, etc.
Outputs	Number of outpatient and emergency visits	The number of patients coming for outpatient and emergency diagnostic services
	Number of discharged patients	The number of discharged patients after hospitalization for various reasons
	Total revenue	Revenue gained from service provision and other activities, including healthcare revenue, drug and medicine sales, financial subsidies, health administrative department subsidies, etc.

Further variables selections were conducted based on a review of international literature on efficiency and productivity research of Chinese hospitals with DEA. In the study of Gai et al. [11], 4 input variables (number of medical staff; number of beds; value of fixed capital; and hospital expenditures) and 3 output variables (outpatient and emergency visits, number of inpatients, and hospital revenue) were applied. We argue that this study has issues in variable selection similar to other Chinese studies: (1) the double counting problem was still unresolved; (2) With both volume and monetary variables, technical efficiency scores not only reflect technical efficiency, but also allocative efficiency, making technical efficiency measurement invalid. In general, we followed the approach of Ng [12] with further improvements. Ng held that labor and capital are both important in delivering health services in hospitals. In her research, the number of physicians, nurses, pharmacists, other medical and administrative staff were used respectively as labor variables [12]. However, the curse of dimensionality is always present in nonparametric estimation [25]. In order to reduce dimensionality, we followed Nuti et al. [26] to merge highly relevant variables of a specific group into one category. Specifically, we used the number of employees as a whole instead of physicians, nurses, technicians, etc. to represent labor input. In terms of capital, Ng [12] used the number of beds by arguing that “a hospital's capital stock was proxied by the number of beds”. However, in China, the number of beds may not reflect the dynamic situation of hospitalization in China because many temporary beds were used. Instead, we adopted “the actual number of open beds” for our study. Unlike most of Chinese literature, we excluded “fixed assets” due to: (1) it is a monetary variable and should be avoided to use in technical efficiency measurement; (2) it is highly relevant to hospital beds and “the actual of open beds” was already chosen to represent capital. However, we still keep the variable for our descriptive analysis.

In terms of output variables, Ng [12] used “the number of outpatient cases” and “the number of inpatient cases”. Besides the number of outpatient visits, we added the number of emergency visits to reflect outputs more accurately. In terms of “bed occupancy rate” and “average length of stay in hospital”, although many Chinese researchers use them in DEA (the average length of stay in hospital was transformed into reciprocal quantity), we hold that simple ratios are inappropriate for application in DEA, as the numerators and/or denominators may be closely related to other variables. We also excluded monetary variables “total expenditure” and “total revenue” to avoid double counting and mixing of technical efficiency and allocative efficiency. As a result, 2 input variables (actual number of open beds, number of employees) and 2 output variables (number of outpatient and emergency visits, number of discharged patients) were selected for our analysis. Still we keep “bed occupancy rate” and “average length of stay in hospital” for our descriptive analysis. These simple ratio variables can be regarded as process indicators instead of direct output variables and may help better understanding efficiency and productivity results.

### Data source and sample

Data from the period 2006–2009 had been collected by the National Institute of Hospital Administration (NIHA), Ministry of Health of P. R. China. NIHA has granted us permission to use these data. As basic requirement to apply DEA method is to select a group of similar units, in the data set 7 specialty hospitals were excluded and 12 third-grade Class A general public hospitals were finally selected.

### Data processing and analysis

Data of some variables such as fixed asset, total expenditure, total revenue were suitably processed to eliminate inflation impact. All data of the 12 hospitals in the 4 years were transformed from excel spreadsheet into a dataset file. Then a descriptive analysis was conducted using Stata 10.0. The data were further transformed into a text file and Malmquist index analysis was applied using DEAP(V2.1) software.

## Results

### Descriptive statistics and analysis

Table 2 contains a descriptive statistics of sample hospitals, showing that the data of all the input variables had grown each year, implying that sample hospitals had experienced capacity and scale development in the 2006–2009 period. These hospitals provided services to more patients and enjoyed higher revenue. Bed occupancy rates were very high, indicating these hospitals exhibited high attending rates over years; the average length of stay in hospital decreased from 13.4 to 11.3 days, indicating significant process improvements.

**Table 2:** Descriptive statistics of 12 hospitals in period 2006-2009

**No.**	**Input and output variables**	**2006**	**2007**	**2008**	**2009**	**Growth rates**
1	Actual number of open beds	1187	1187	1249	1280	7.83%
2	Number of employees	2642	2778	2968	2939	11.24%
3	Fixed asset	92135	101988	113190	120400	30.68%
4	Total expenditure	90296	116934	112169	116928	29.49%
5	Number of outpatient and emergency visits	1210433	1456612	1615103	1721248	42.20%
6	Number of discharged patients	30099	32648	36059	39579	31.50%
7	Total revenue	88970	118321	141699	148283	66.67%
8	Bed occupancy rate	0.94	0.97	0.95	0.95	1.06%
9	Average length of stay in hospital	13.4	12.9	12.1	11.3	-15.67%

**Figure 1:**
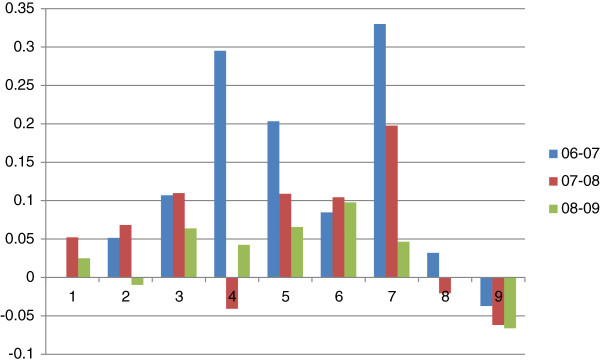
Annual growth rates of 9 variables.

Figure 1 is about annual growth rates of 9 variables for two consecutive years. Although the actual number of open beds and the number of employees had not risen sharply, fixed assets and total expenditure had grown rapidly. In contrast, the number of outpatient and emergency visits, the number of discharged patients and total revenue had grown much faster than other inputs, indicating improvements in productivity. It is surprising that growth rate of total revenue was highest, indicating hospitals’ profit-seeking behaviors may influence their delivery of health services.

### Malmquist index analysis

**Figure 2:**
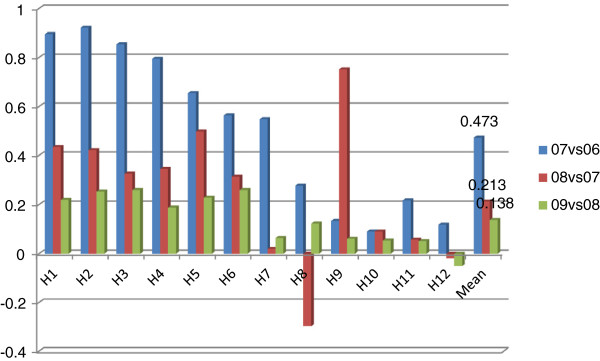
TFP changes for two consecutive years.

**Figure 3:**
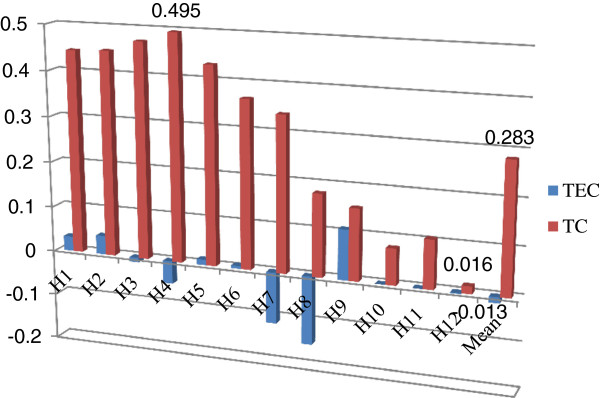
Decomposition of TFP into technological change and technical efficiency change.

**Figure 4:**
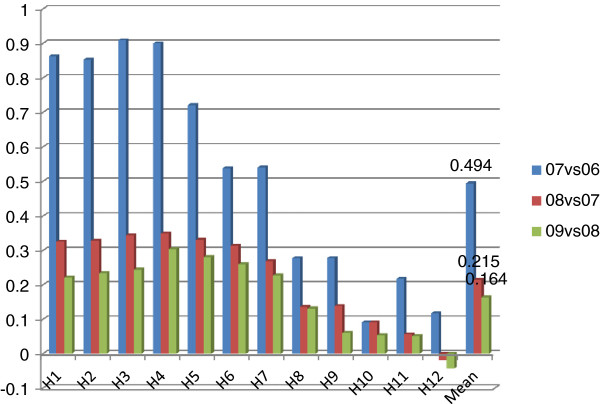
Technological changes for consecutive years.

**Figure 5:**
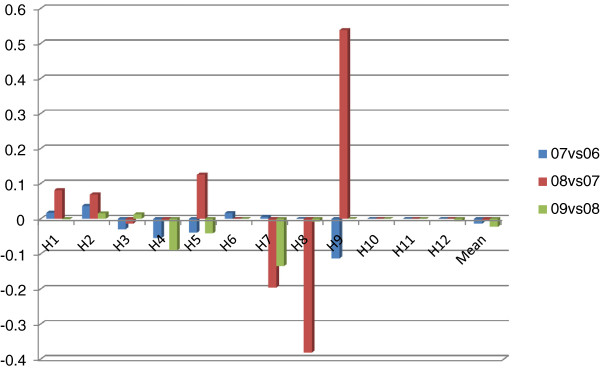
Technical efficiency changes for two consecutive years.

**Figure 6:**
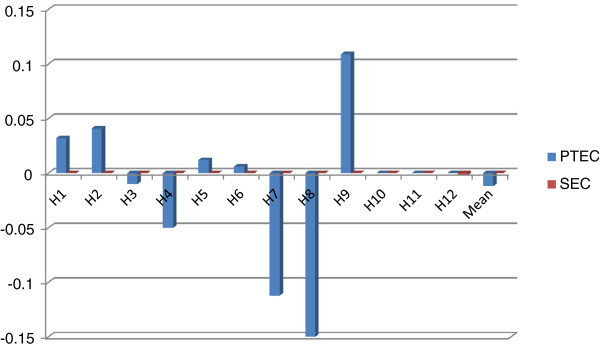
Decomposition of technical efficiency into pure technical efficiency change and scale efficiency change.

**Figure 7:**
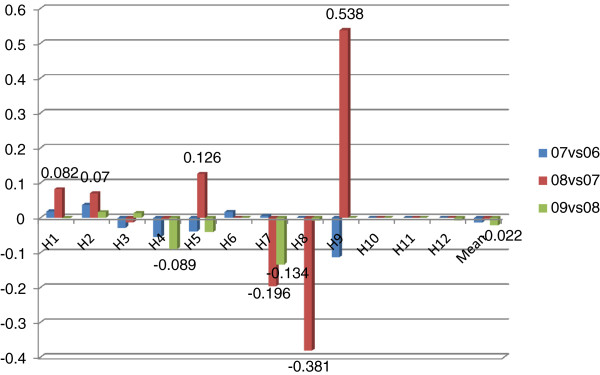
Pure technical efficiency changes for two consecutive years.

**Figure 8:**
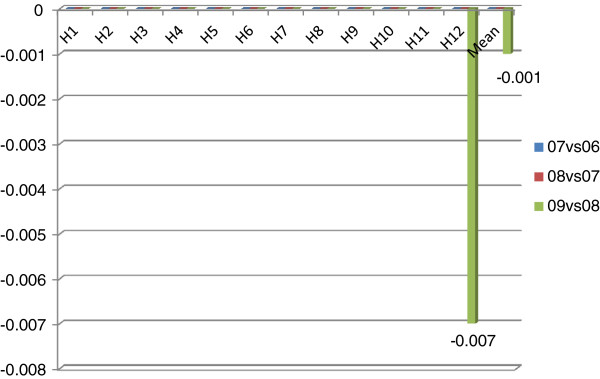
Scale efficiency changes for two consecutive years.

In order to further explore the driving factors behind improvements in productivity, Malmquist index analysis was applied and results are presented in Figures 2–8.

### TFP Changes

Figure 2 is about TFP changes for two consecutive years in the 2006–2009 period. In general, average annual TFP growth rate of sample hospitals was 26.7%, ranging from 0.3% to 50.8%. All hospitals had experienced productivity growth. However, for most, TFP growth rates had dramatically declined over years, calling for effort to maintain TFP growth. Moreover, some hospitals had surprising growth rates while others had limited growth rates, indicating that some hospitals can still put effort to further foster their TFP growth.

Figure 3 is a decomposition of the TFP into technological change and technical efficiency change. Technological change had average annual contribution of 28.3% to the growth rates, ranging from 1.6% to 49.5%. Some of the hospitals still have potential to further increase the contribution of technological change to productivity. Differently from technological change, sample hospitals had been stagnated in technical efficiency. Some even had decline in technical efficiency.

### Technological changes

Figure 4 is about technological changes for two consecutive years. Similar to TFP growth, the growth rates of technological changes had dramatically declined over years, indicating technological change needs to be further consolidated. On the other side, some hospitals had very limited or even negative growth in technological change, reflecting that these hospitals still have untapped potential for improvements.

### Technical efficiency changes

Figure 5 is about technical efficiency changes for two consecutive years. In the year 2006–2007, only 4 hospitals had improvements and the growth rates were low; 4 did not exhibit any change; 4 had declines. In the year 2007–2008, only 4 hospitals showed improvements and growth rates varied; 4 did not exhibit any change; 4 had declines. In the year 2008–2009, only 2 hospitals showed improvements and growth rates were trivial; 4 did not exhibit any change; 6 had a decline. For each year, almost 1/3 of the hospitals had improvements in technical efficiency; 1/3 did not exhibit any change; 1/3 had declines.

Figure 6 is a decomposition of technical efficiency into pure technical efficiency change and scale efficiency change. In the 2006–2009 period, 5 hospitals had improvements in pure technical efficiency; 3 had neither improvements nor declines, 4 had declines in pure technical efficiency. In terms of scale efficiency, 11 hospitals had stagnation; 1 hospital had a slight decline.

Figure 7 is about pure technical efficiency changes for two consecutive years. In both the year 2006–2007 and the year 2007–2008, 4 hospitals had improvements in pure technical efficiency; 4 had neither improvements nor declines, 4 had declines. In the year 2008–2009, only 2 hospitals had improvements; 5 hospitals had neither improvements nor declines; 5 hospitals had declines.

Figure 8 is about scale efficiency changes for two consecutive years. In both the year 2006–2007 and the year 2007–2008, all sample hospitals had neither improvements nor declines in scale efficiency. In the year 2008–2009, 2 hospitals had slight declines; others had stagnated. It can be asserted that all the samples had stuck in scale efficiency.

## Discussion

### Technological changes

Our results indicate that in the 2006–2009 period, substantial technological change was the largest contributor to productivity growth among sample hospitals in Beijing, which is consistent with the findings of Pang & Wang [6] and Ng [12]. In contrast, the contribution of technological change to TFP growth in Beijing is the highest, which may indicate that Beijing enjoys a more advantageous position to maintain technological advantage, which can help understanding why most of Chinese terminally ill patients would always choose to go to best hospitals in Beijing for their last chances of treatments.

In sample hospitals, although the actual number of open beds (7.83%) and the number of employees (11.24%) did not rise sharply, the fixed assets had grown by 30.7%, which mean that hospitals still managed to expand their capacity and scale by increasing more fixed assets, such as building more facilities, purchasing more high-tech equipments, etc. As a result, the total revenue had grown by 66.67% in the same period. Such finding, to some extent, is consistent with the assertion of Ng [12] that the observed productivity growth rates from 68% to 94% was resulted from adoption of high-tech treatments in hospitals. There are many factors contributing to this phenomenon: (1) sample hospitals could only get less than 10% subsidies from government funding and were allowed to survive by selling drugs and by providing physician examinations, which in return led to over-prescribing drugs and tests, adopting high-tech treatments, and delivering unnecessary medical care, etc.; (2) the prices for health services provided have long been twisted among Chinese hospital system. Many health services provided e.g. labor have been charged at low prices under cost. However, hospitals can have higher margins from services provided by high-tech equipments, which is a driving factor to increase their number of high-tech equipments; (3) there lacks of specific laws, rules and regulations on how hospitals can be allowed to manage their profits for their own development. If they have large profits and keep them in banks, government will be reluctant to provide additional subsidies. Instead, being in debt renders “more reasonable” to ask for subsidies. Therefore, expanding capacity and scale is hospitals’ rational choice. In order to recoup their investment, hospitals would still resort to make profits on patients if effective monitoring is absent. According to Yip and Hsiao [27], providers’ profit seeking behaviors will result in waste and inefficiency within the healthcare system itself.

In our results, the growth rates of technological change had dramatically declined each year. Although the adoption of new drugs and medicine can facilitate the cure of difficult and complicated illness of patients and help improve productivity, it is obvious that most of these driving factors do not have cost constraints, which will necessarily result in increased healthcare expenses. It is evidenced in the United States that technological progress is a large contributor to high healthcare spending [28]. More driving factors such as process innovation, new treatment methods, and so on need to be explored. In the meanwhile, more hospital employees need to be trained to increase their expertise. Government, universities and hospitals can collaborate closely to cultivate more graduates for hospitals.

### Technical efficiency changes

In our findings, technical efficiency changes had trivial contributions to TFP growth, which is consistent with the findings of most of Chinese researchers, indicating this is a common problem among Chinese public hospitals.

In particular, no hospitals showed improvements in scale efficiency, which may be explained by the fact that fixed assets had grown much faster than the number of employees, indicating more qualified health workers were needed to fill vacancies resulting from capacity and scale expansion. However, hospitals were lack of driving factors to cultivate their own staff and they began competing healthcare professionals against each other. As the whole supply is limited, the flow of these workers from first and second grade hospitals to third-grade hospitals would reduce productivity of first two. Therefore, while governments of all levels are restraining big hospitals’ inclination for larger scales, relevant policies should be in place to encourage them to cultivate more qualified staff to satisfy the needs resulting from over-expansion of hospital capacity and scale. Only when hospitals have sufficient qualified healthcare staff will they be able to maximize scale efficiency.

Other aspects causing stagnation or decline in technical efficiency can be classified as pure technical efficiency problems, which call for better hospital governance, better hospital management, etc. In terms of hospital governance, each sample hospital had multiple governing bodies, which increased coordination and bureaucracy; there lacks of well designed mechanisms, rules and regulations to select competitive directors and clearly define their rights, interests, responsibility and accountability; the limited rights of hospital directors also constrain their abilities to hire, fire, pay, and promote employees, which would reduce the quality of management practices and therefore influence pure technical efficiency. In terms of hospital management, Bloom et al. [29] held that product market competition, labor regulation, ownership, education, information etc. can help explain management practices within countries or across industries. All these challenges call for deeper hospital reforms to improve hospital governance and hospital management and thus improving pure technical efficiency.

### Allocative efficiency

Farrell [30] divided overall efficiency into technical efficiency and allocative efficiency. Achieving technical efficiency does not necessary imply allocative efficiency will be met. In our study, as we lack of sufficient price information, we focus on measurement and analysis of inputs and outputs in volume terms. In this way, allocation efficiency cannot be measured. However, some actions can still be made to improve allocative efficiency.

First, the tasks of hospital directors and managers should be separated from clinical activities. The nature of bureaucratic tasks of hospital management detracts away from patient care time [31]. It has been common in China that hospital directors and managers not only are engaged in managerial work, but also provide clinical services directly to patients. It would be highly inefficient and costly to transform an excellent doctor to be a director or a manager, since it takes a long time and huge resources to cultivate an expert doctor, especially when hospitals lack of expert doctors due to their over-expansion in capacity and scale. Therefore, directors and managers not participating in clinical activities would improve allocative efficiency.

Second, new provider payment methods and mechanisms can be explored. In sample hospitals, payment methods were all based on fee-for-service. In Beijing, DRG and other mixed provider-payment methods have been introduced and experimented [32,33], which have demonstrated preliminary advantages to save cost and to reduce waiting time (improvement in efficiency). Government of all levels can enlarge the coverage and find ways to consolidate these achievements, such as providing more funding and policy supports to encourage more volunteer hospitals to apply for new payment methods and mechanisms, which will help improving allocative efficiency.

Third, relevant laws and regulations are required to enable the differentiation of healthcare services provided. International experience suggests that countries where primary care physicians act as gatekeepers are more efficient than countries without gatekeepers [34]. Although Chinese government is applying different reimbursement rates between community health service centers and different levels of hospitals as a way to guide inhabitants to go first to community health service centers, this differentiation cannot be accomplished alone without establishing and implementing relevant government laws, rules and regulations. By differentiating services provided, the proportions of human resource inputs will be greatly optimized and less healthcare cost will be spent with the optimized inputs, improving both technical efficiency and allocative efficiency.

### The need for multi-dimensional performance evaluation

It is insufficient for government to use efficiency measurement for policy decision-making, as efficiency is just one dimension to drive performance, while effectiveness, appropriateness, clinical quality, patient safety, financial equilibrium etc. are all relevant to the performance of a healthcare system [35]. This calls for introduction of a multi-dimensional reporting measurement system. At the end of July 2011, Beijing established a hospital authority within the municipal Bureau of Health to manage 22 municipal third-grade Class A hospitals, 7 of which hospitals are our sample hospitals. It will be of strategic importance if the hospital authority can enable performance benchmarking among their affiliated hospitals, which is both an effective way to improve efficiency and performance and an effective tool to learn best practices from peer hospitals. In this aspect, the experience in Tuscany region in Italy is a good example for China’s purpose [36-38] and Li et al. [39] explored the possibility of building China’s municipal healthcare performance evaluation system by learning from the Tuscan experience.

### Limitations and further development

•This research has some limitations. The first one is the limited number of hospitals available for the research. In further research design, the sample can be extended to include all three grades of hospitals in Beijing or in another region, with respective efficiency and productivity analysis for comparison. In this way, the flow of healthcare resources among the three types of hospitals would be much clearer, which would be helpful to identify weakness of hospital system for further improvements in efficiency and productivity.

•In our study, bias adjustments of efficiency scores were not conducted due to limitation of Coelli’s approach. In future research, a Bootstrap-Malmquist approach can be applied for more exact results.

•Coelli and Rao [40] pointed out that in Malmquist DEA, the explicit price information is replaced by implicit (or shadow) price information. These implicit prices may differ substantially from market prices, thus result in TFP measures that may differ substantially from those obtained using other methods. Furthermore, the piece-wise linear nature of the DEA surface (and the regular occurrence of slack regions) can result in wide variations in shadow prices, which subsequently lead to significant differences in the weights assigned to different inputs across the sample. Therefore, in future research, alternative methods are encouraged to be applied for robustness check.

## Conclusions

In our preliminary study, third-grade Class A public hospitals in Beijing (2006–2009) experienced rapid growth rates in TFP, which mainly resulted from substantial technological progress, while technical efficiency represented by pure technical efficiency and scale efficiency had a slight decline or stagnation. Both government and hospitals need significant reforms and transitions to improve technological progress, technical efficiency and allocative efficiency to further increase growth rates of efficiency and productivity of public hospitals.

In our study, two improvements have been made in the methodology compared with most Chinese researchers in efficiency and productivity measurement of Chinese hospitals. The first is that inflation effect was excluded from monetary variables. The second is that ratio indicators and monetary variables were not used together with volume variables in DEA. However, due to limitations of our preliminary study, more empirical studies are needed to include more hospitals of all grades and more alternative methods are encouraged to be used for robustness checks.

## Abbreviations

TFP: Total Factor Productivity; OE: Overall Efficiency; AE: Allocative Efficiency; TE: Technical Efficiency; DEA: Data Envelopment Analysis; DMU: Decision-making Unit; SFA: Stochastic Frontier Analysis; TC: Technological Change; TEC: Technical Efficiency Change; SEC: Scale Efficiency Change; NIHA: National Institute of Hospital Administration, P.R. China

## Competing interests

There are no competing interests among the co-authors.

## Authors’ contributions

SPD has developed the primary framework of this research; HL has written the final manuscript after making substantial improvements by strengthening each section of the paper and taking inflation into consideration; TFL has strengthened the parts concerning hospital accreditation and hospital scale. All authors read and approved the final manuscript.

## Pre-publication history

The pre-publication history for this paper can be accessed here:

http://www.biomedcentral.com/1472-6963/14/158/prepub

## References

[B1] Chen H Ding JT Health investment structure, health improvement and economic growth J Public Manage 2010 14 54 62

[B2] Wang H Xu T Xu J Factors contributing to high costs and inequality in China’s health care system JAMA 2007 14 1928 1930 10.1001/jama.298.16.1928 17954544

[B3] Ministry of Health of China Ministry of Health of China: The rules for accreditation management of general hospitals (Pilot draft) Government document 1989 Available at http://www.orthochina.com/orthopedic/topic.jsp?className=%BD%A1%BF%B5%BD%CC%D3%FD&ID=4605

[B4] Liu TF Institutional change and path selection of hospital accreditation in China Chin Health Qual Manage 2011 14 31 34

[B5] Kuang L The continuing scale expansion mechanism of Chinese public hospitals Chin J Health Policy 2011 14 28 37

[B6] Pang HM Wang XW Evaluating efficiency of level III general hospitals in China based on DEA Malmquist index Chin Hosp Manage 2010 14 35 37

[B7] Wang H Ma Y Li B Li DN Application of DEA in evaluating the efficiency of third-grade hospitals in Harbin Chin J Hosp Stat 2006 14 289 292

[B8] LU WJ Yang Q Feng ZC Analysis of Malmquist index of the dynamic changes of hospital efficiency in Wuhan Chin Hosp Manage 2011 14 28 31

[B9] Wang W Chen SX Peng XM Zhai ZT Chen SR Tian YL Efficiency analysis of public hospitals in Guangdong Province Chin Hosp Manage 2008 14 16 19

[B10] Liu J He MQ Research on the assessment of technical efficiency of comprehensive hospitals and determining factors Sci Manage Res 2010 14 69 71

[B11] Gai RY Zhou CC Xu LZ Zhu M Wang XZ Li SX Zheng WG Song PP Yang XL Fang LY Zheng YC Tang W Health resources allocation and productive efficiency of Chinese county hospitals: data from 1993 to 2005 BioScience Trends 2010 14 218 224 21068473

[B12] Ng YC The productive efficiency of Chinese hospitals China Econ Rev 2011 14 428 439 10.1016/j.chieco.2011.06.001

[B13] Worthington AC Frontier efficiency measurement in health care: a review of empirical techniques and selected applications Med Care Res Rev 2004 14 135 170 10.1177/1077558704263796 15155049

[B14] Hollingsworth B The measurement of efficiency and productivity of healthcare delivery Health Econ 2008 14 1107 1128 10.1002/hec.1391 18702091

[B15] Daraio C Simar L Advanced robust and nonparametric methods in efficiency analysis. Methodology and Applications 2007 New York: Springer

[B16] Charnes A Cooper W Rhodes E Measuring the efficiency of decision making units Eur J Oper Res 1978 14 429 444

[B17] Banker RD Charnes A Cooper W Some models for estimating technical and scale inefficiencies in data envelopment analysis Manage Sci 1984 14 1078 1092

[B18] Wheelock DC Wilson PW Technical progress, inefficiency, and productivity change in U.S. banking, 1984–1993 J Money Credit Bank 1999 14 212 234 10.2307/2601230

[B19] Malmquist S Index numbers and indifference surfaces Trabajos de Estadistica 1953 14 209 242 10.1007/BF03006863

[B20] Caves DW Christensen LR Diewert WE Multilateral comparisons of output, input, and productivity using superlative index numbers Econ J 1982 14 73 86 10.2307/2232257

[B21] Lovell CAK The decomposition of Malmquist productivity indexes J Prod Anal 2003 14 437 458 10.1023/A:1027312102834

[B22] Fare R Grosskopf S Norris M Zhang ZY Productivity growth, technical progress, and efficiency change in industrialized countries Am Econ Rev 1994 14 66 83

[B23] Moffat BD Valadkhani A Harvie C Malmquist indices of productivity change in Botswana’s financial institutions Glob Bus Econ Rev 2009 14 28 43 10.1504/GBER.2009.025380

[B24] Coelli T A guide to DEAP(V2.1): A data envelopment analysis (computer) program 1996 Armidale: University of New England

[B25] Simar L Wilson PW Sensitivity analysis of efficiency scores: how to bootstrap in nonparametric frontier models Manage Sci 1998 14 49 61 10.1287/mnsc.44.1.49

[B26] Nuti S Daraio C Speroni C Vainieri M Relationships between technical efficiency and the quality and costs of health care in Italy Int J Qual Health C 2011 14 324 330 10.1093/intqhc/mzr005 PMC3092690 21454349

[B27] Yip W Hsiao W China’s health care reform: a tentative assessment China Econ Rev 2009 14 613 619 10.1016/j.chieco.2009.08.003

[B28] OECD Health at a Glance 2011: OECD Indicators. Report 2011 Paris

[B29] Bloom N Van Reenen J Why do management practices differ across firms and countries? J Econ Perspect 2010 14 203 224 10.1257/jep.24.1.203

[B30] Farrell MJ The measurement of productive efficiency J Roy Stat Soc A Gen 1957 14 253 290 10.2307/2343100

[B31] The NHS Confederation Challenging bureaucracy 2013 London: Report

[B32] Gao W DRG pilot experiment in Beijing Chin Hosp CEO 2011 14 32 39

[B33] Hu B Ji XM Fei XL Wei L Wang RX Analysis of the medical services and payment mode for patients with emergency in a Beijing Hospital from 2007 to 2008 Chin Hosp Manage 2010 14 24 26

[B34] Vasanthakumar NB Institutional arrangements and efficiency of health care delivery systems Eur J Health Econ 2005 14 215 221 10.1007/s10198-005-0294-1 15864675

[B35] Smith PC Mossialos E Papanicolas I Leatherman S Performance measurement for health system improvement: experiences, challenges and prospects European Observatory on Health Systems and Policies 2009 Cambridge University Press

[B36] Nuti S Vainieri M Bonini A Disinvestment for re-allocation: a process to identify priorities in healthcare Health Policy 2010 14 137 143 10.1016/j.healthpol.2009.11.011 20015568

[B37] Nuti S Seghieri C Vainieri M Assessing the effectiveness of a performance evaluation system in the public health care sector: some novel evidence from the Tuscany region experience J Manage Gov 2013 14 59 69 10.1007/s10997-012-9218-5

[B38] Nuti S Seghieri C Vainieri M Zett S Assessment and improvement of the Italian healthcare system: first evidences from a pilot national performance evaluation system J Healthc Manag 2012 14 182 198 22724376

[B39] LI H Barsanti S Bonini A Building China’s municipal healthcare performance evaluation system: a Tuscan perspective Int J Qual Health C 2012 14 403 410 10.1093/intqhc/mzs032 22687705

[B40] Coelli TJ Rao DSP Implicit Value Shares in Malmquist TFP Index Numbers. Centre for Efficiency and Productivity Analysis CEPA Working Papers No. 4/2001 University of New England

